# The UCLA multimodal connectivity database: a web-based platform for brain connectivity matrix sharing and analysis

**DOI:** 10.3389/fninf.2012.00028

**Published:** 2012-11-28

**Authors:** Jesse A. Brown, Jeffrey D. Rudie, Anita Bandrowski, John D. Van Horn, Susan Y. Bookheimer

**Affiliations:** ^1^Center for Cognitive Neuroscience, University of California Los AngelesLos Angeles, CA, USA; ^2^Department of Psychiatry and Biobehavioral Sciences, University of California Los AngelesLos Angeles, CA, USA; ^3^Interdepartmental Program in Neuroscience, University of California Los AngelesLos Angeles, CA, USA; ^4^Brain Mapping Center, University of California Los AngelesLos Angeles, CA, USA; ^5^Center for Research in Biological Systems, University of California San DiegoSan Diego, CA, USA; ^6^Laboratory of Neuroimaging, Department of Neurology, University of California Los AngelesLos Angeles, CA, USA

**Keywords:** graph theory, data sharing, functional connectivity, structural connectivity, resting-state fMRI, diffusion-weighted MRI

## Abstract

Brain connectomics research has rapidly expanded using functional MRI (fMRI) and diffusion-weighted MRI (dwMRI). A common product of these varied analyses is a connectivity matrix (CM). A CM stores the connection strength between any two regions (“nodes”) in a brain network. This format is useful for several reasons: (1) it is highly distilled, with minimal data size and complexity, (2) graph theory can be applied to characterize the network's topology, and (3) it retains sufficient information to capture individual differences such as age, gender, intelligence quotient (IQ), or disease state. Here we introduce the UCLA Multimodal Connectivity Database (http://umcd.humanconnectomeproject.org), an openly available website for brain network analysis and data sharing. The site is a repository for researchers to publicly share CMs derived from their data. The site also allows users to select any CM shared by another user, compute graph theoretical metrics on the site, visualize a report of results, or download the raw CM. To date, users have contributed over 2000 individual CMs, spanning different imaging modalities (fMRI, dwMRI) and disorders (Alzheimer's, autism, Attention Deficit Hyperactive Disorder). To demonstrate the site's functionality, whole brain functional and structural connectivity matrices are derived from 60 subjects' (ages 26–45) resting state fMRI (rs-fMRI) and dwMRI data and uploaded to the site. The site is utilized to derive graph theory global and regional measures for the rs-fMRI and dwMRI networks. Global and nodal graph theoretical measures between functional and structural networks exhibit low correspondence. This example demonstrates how this tool can enhance the comparability of brain networks from different imaging modalities and studies. The existence of this connectivity-based repository should foster broader data sharing and enable larger-scale meta-analyses comparing networks across imaging modality, age group, and disease state.

## Introduction

Successful neuroimaging data sharing efforts have taken a variety of organizational approaches, including top-down centralized strategies and bottom-up grassroots efforts. Centralized projects such as the Alzheimer's Disease Neuroimaging Initiative (ADNI; http://www.adni-info.org) begin by defining a targeted subject population, the type of imaging data to be included, and a set of criteria to ensure the quality and similarity of the data collection across multiple sites and scanners. Grassroots projects like the International Neuroimaging Datasharing Initiative (INDI; http://fcon_1000.projects.nitrc.org/index.html) are less restrictive and encourage the broad sharing of data across centers, subject pools, and scan types. Once data has been collected, it can be stored in a database where users can search and download desired data. This allows researchers to freely access the data, enabling them to apply their own preprocessing and run custom analyses. These sites typically collect image files in a specific format such as NiFTI or DICOM along with relevant meta-information about the data acquisition, the individual receiving the scan, and the study design.

Another variety of neuroimaging databases store processed data and/or analysis results. The BrainMap database (http://brainmap.org) stores stereotaxic standard-space coordinates of activation peaks from fMRI and PET data analyses and associated metadata including the number of subjects, the subject disease state (healthy or diseased), the applied analysis techniques, the experimental paradigm, and the cognitive process under investigation (e.g., working memory) (Fox and Lancaster, 2002; Laird et al., 2005). SUMS-DB is a database for sharing structural and functional brain mapping study results, also based on stereotaxic coordinates (http://sumsdb.wustl.edu). These databases foster meta-analyses by compiling findings across studies into a common coordinate space, allowing users to probe for findings within a specific brain region or network.

In between the extremes of stereotaxic foci and raw data repositories exist many intermediates of “processed” neuroimaging data. Processed data can be beneficial in a shared data setting because it requires less analysis by subsequent users than raw data, while enabling more thorough re-analysis than a set of significant spatial coordinates does. One example of processed neuroimaging data that has been particularly useful in explaining brain connectivity properties is the “connectivity matrix” (CM). A typical connectivity analysis in a neuroimaging study measures the strength of connection between different brain regions. Connection strength can be defined in a variety of ways. In functional MRI (fMRI), the statistical correlation of BOLD intensity changes in two regions is commonly used as a measure of “functional connectivity.” In diffusion tensor imaging (DTI) and related diffusion-weighted MRI (dwMRI) modalities, the density of axonal bundles or “structural connectivity” between two regions can be quantified using fiber tractography methods. For neuroimaging experiments whose field of view is sufficiently large to cover the entire brain, one can determine the whole brain connectivity “graph” by portioning the brain into constituent regions and determining the direct connectivity between every pair of regions. In this graph representation of connectivity, the pattern of connections between nodes is stored in a CM where rows/columns in the matrix represent brain regions (nodes) and the matrix cell where these two regions intersect stores the connection strength between the two regions (edges).

Graph theoretical analyses can be performed on a CM in order to characterize a network's global integration, local interconnectivity, modularity, cost efficiency, and robustness to lesioning (Bullmore and Sporns, 2009; Sporns, 2010). Analyses of CMs derived from structural and functional neuroimaging modalities have led to the recognition of a core set of structural hubs in the posterior cingulate and precuneus (Hagmann et al., 2008); the determination that functional network hubs coincide with the sites of greatest amyloid deposition in Alzheimer's Disease (Buckner et al., 2009); and the discovery that flexible reconfiguration of functional connectivity modules is critical for motor learning (Bassett et al., 2011b). CMs have been derived from structural and fMRI data from the same subjects in several studies, indicating moderate correspondence of the structural and functional connectivity strength between regions (Honey et al., 2007, 2009; Hagmann et al., 2010).

Connectivity matrices are a highly distilled representation of brain connectivity. Despite this reduction, they contain sufficient information to capture individual characteristics such as age (Dosenbach et al., 2010), gender (Yan et al., 2011), intelligence quotient (IQ) (Li et al., 2009; van den Heuvel et al., 2009b), and disease state (Supekar et al., 2008; Craddock et al., 2009; Lo et al., 2010). Graph theory adds to the utility of a CM by quantifying how brain regions are integrated into a global unit, rather than how they act in isolation. A great deal of research effort and funding has been dedicated to describing the human connectome, which is at its essence a CM (Sporns et al., 2005). CMs are therefore an ideal product to compile and share with the community. Here we present the UCLA Multimodal Connectivity Database (UMCD henceforth; http://umcd.humanconnectomeproject.org), a website that allows CMs and meta-information to be uploaded and shared with the public. It provides a dynamic, sortable search engine for locating relevant datasets. It also provides a platform for graph theory analysis of any publicly shared CM, reporting basic graph properties, graph theoretical metrics, and interactive 3D/2D visualizations.

## Materials and methods

### The UCLA multimodal connectivity database

**Figure 1:**
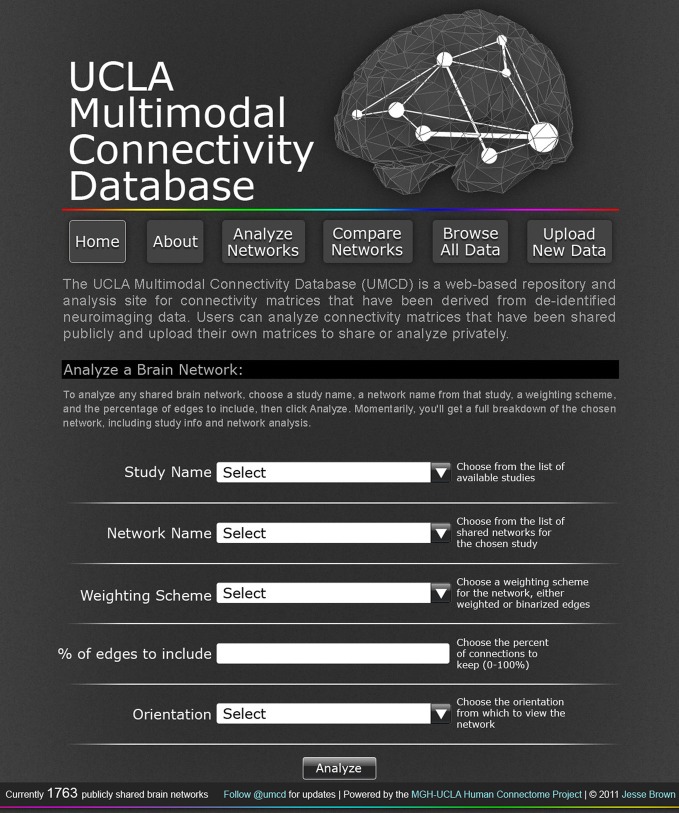
**The UCLA Multimodal Connectivity Database homepage, where a user can configure the analysis for any CM publicly shared on the site.** The user can select from any of the studies for which data has been publicly shared on this site with the “Study Name” dropdown menu. Once a study is selected, all the individual brain networks that have been shared for that study will appear in the “Network Name” dropdown menu. After selecting an individual network to analyze, the user must specify a “Weighting scheme” and “% of edges to include” for the analysis, choose an orientation in which to render the analysis-based network images, and click “Analyze.”

**Figure 2:**
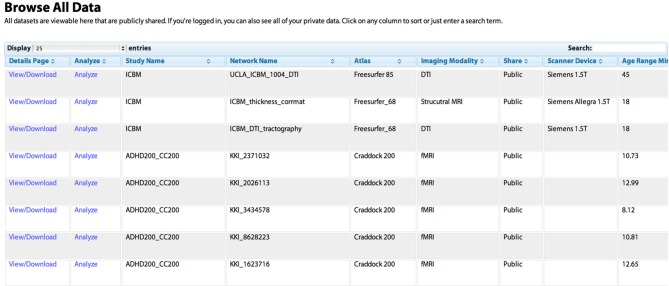
**The “Browse All Data” page.** Studies are initially listed in the order in which they were shared by users. Any column can be sorted by clicking on the heading, allowing for example the grouping of all DTI studies, or the sorting of networks based on the age of the subject. The “Search” field can be used to dynamically constrain which records from the database are shown. The “View/Download” link takes the user to a “profile” page for the individual network that contains more detailed information (see Figure 5). The “Analyze” link takes the user to the “Analyze Network” page with the “Study Name” and “Network Name” pre-selected, allowing the user to run a network analysis simply by clicking “Analyze.”

**Figure 3:**
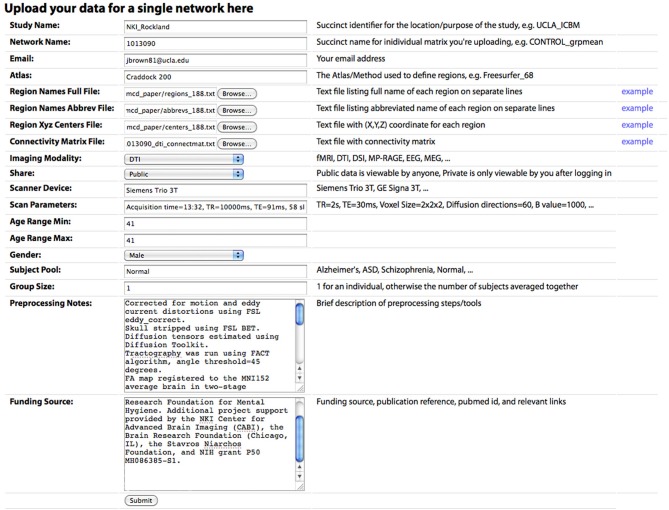
**The “Upload New Data” page, where a user can upload a CM.** Descriptions of each field are included in the main text.

The UMCD is a public website found at http://umcd.humanconnectomeproject.org. The site has five main options:

1. “Analyze a network,” where any publicly shared network can be selected, analysis parameters can be configured, and the network analysis is performed on the site (Figure 1). Once the analysis is complete, the user is redirected to a Results page (contents described below)
2. “Compare networks,” similar to “Analyze” but allows the user to select two networks to compare side by side
3. “Lesion a network,” similar to “Analyze,” with the additional option to select any subset of regions in the chosen network to virtually “lesion,” setting all connections from the selected nodes to zero; the results of the analysis for the unlesioned and lesioned versions of the network are displayed side by side
4. “Browse networks,” allows the user to view all available networks and keyword search for specific datasets or sort all datasets based on different criteria (Figure 2)
5. “Upload a network,” where the user can upload data, either to share with the public or to keep private but compare to public data (Figure 3).

The UMCD requires users to register with an email address and a password in order to share data. Once an account has been created, the user has the option to share data Publicly, in which case any site visitor can analyze or download the data, or Privately, allowing only the user to access this data when they are logged in.

#### Design

The UMCD is built with the web2py framework (http://web2py.com). This Python-based framework uses the Model-View-Controller (MVC) architecture. This enables the seamless coupling of HTML pages with Python code and libraries for performing data analysis and visualization. The site uses a MySQL (http://www.mysql.com) database to store all data including user account information and shared data. The NetworkX Python library is used for all graph theory analyses (http://networkx.lanl.gov). This open source library has excellent documentation, an active community, and the ability to easily create network-based visualizations. These visualizations are rendered by passing custom NetworkX Graph objects from NetworkX to matplotlib (http://matplotlib.sourceforge.net), an extensive library for creating data visualizations in Python. All mathematical and statistical calculations use the numpy (http://numpy.scipy.org) and scipy (http://www.scipy.org) libraries.

#### Analysis

On the analysis page, the user can select any “Study Name” for which data has been shared. Once a Study Name has been selected, the individual connectivity matrices associated with that study name will become selectable in the “Network Name” dropdown. The user can select any Network Name. To conduct the network analysis, the user selects a Network Name and then must specify two variables: the Weighting Scheme, which can be binary (the default option) or weighted, specifying the % of edges to include, which can be any integer value between 0 and 100 (20 is the default option). An additional variable, Orientation, dictates the imaging plane in which the network figures will be rendered: axial (default), sagittal, or coronal view. Once all options are specified, the user clicks the Analyze button to run the network analysis.

**Figure 4:**
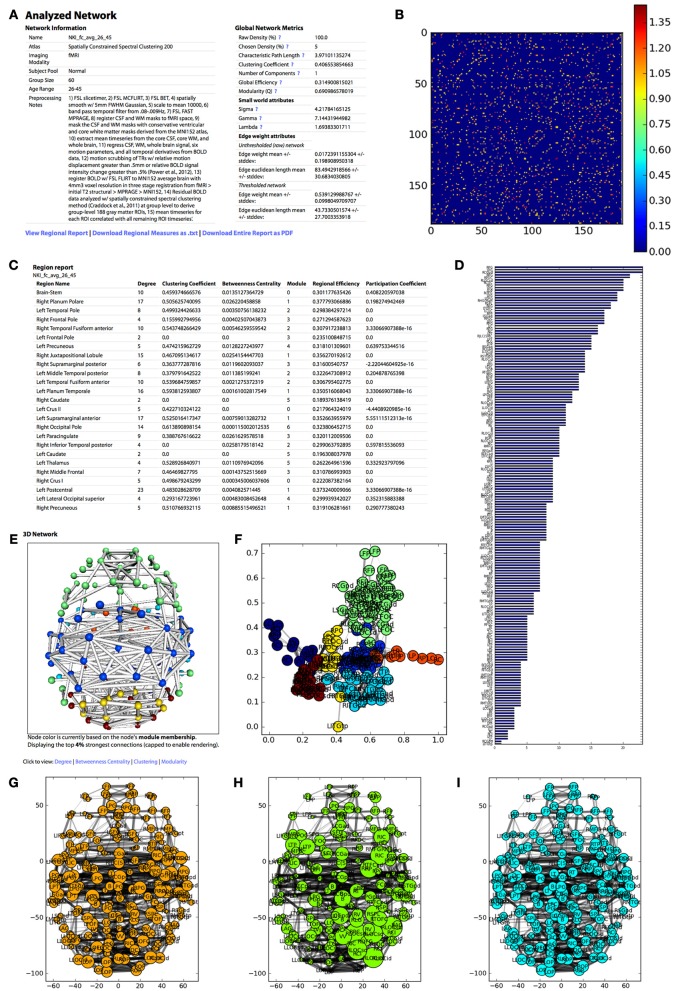
**The appearance of the UMCD report based on the analysis of the NKI fcMRI group-average network, thresholded at 5% with weighted edges. (A)** The meta-information on the network and the global network metrics, including basic edge statistics, graph theory measures, and edge length measures, **(B)** the CM after thresholding, **(C)** the region report, listing the graph theory measures for each node, **(D)** the bar plot of node degree for each node, **(E)** the interactive 3D network rendering from a top view with node color indicating module membership, **(F)** 2D network plot with nodes laid out using the Fruchterman–Reingold force-directed algorithm, with node color indicating module membership, **(G)** the 2D network plot from the top/axial view, with node radius indicating node degree, **(H)** 2D network plot with node radius indicating betweenness centrality, **(I)** 2D network plot with node radius indicating clustering coefficient.

The analysis can take between 10 s to 3 min depending on the size of the network, the weighting scheme, and the threshold. When the analysis is complete, the results are displayed on the Analyzed Network page (Figure 4).

Meta-information for the network that has been analyzed appears at the top of the page and includes the Atlas (i.e., parcellation scheme), Imaging Modality, Subject Pool, Group Size, Age Range, Preprocessing Notes, and Funding Information. Next, a table displays the following Global Network Metrics: the Raw Connection Density (%), the Chosen Density (%), Characteristic Path Length, Mean Clustering Coefficient, Number of Components, Global Efficiency, Modularity (Q), and the small world attributes Gamma, Lambda, and Sigma. Each metric contains a tooltip with a brief description and a link to the NetworkX function used to calculate the measure or the reference from which the formula was taken. For a more complete description of the graph measures calculated on the UMCD, the reader is referred to (Bullmore and Sporns, 2009; Rubinov and Sporns, 2010; Sporns, 2010). For each edge in the raw and thresholded networks, the edge weight mean and standard deviation are shown, along with the edge Euclidean length mean and standard deviations.

The report contains a link to “View Regional Report” where the following nodal network measures appear in a table: full region name, degree, clustering coefficient, betweenness centrality, module membership, regional efficiency, and participation coefficient. These measures can also be downloaded by clicking “Download Regional Measures as.txt” which links to a tab-delimited text file containing all of these metrics. This file can be easily loaded into Matlab or other statistical software in order to perform offline statistical analysis.

The network analysis report includes both three-dimensional and two-dimensional visualizations. The 3D network view is an interactive rendered ball-and-stick model of the network implemented using WebGL (Figure 2). The rendering engine is a modified version of the ChemDoodle Web Components javascript library (http://web.chemdoodle.com). The center of mass for each node in the network appears as a sphere whose radius corresponds to the specific network metric that is selected: degree, betweenness centrality, clustering coefficient, regional efficiency, or participation coefficient. For modularity, all radii are equal and the node color indicates module membership. Each non-zero connection in the network is shown as a cylinder directly connecting the two nodes, whose radius is constant (= 1) for a binary network analysis and is scaled for a weighted analysis. The number of displayed edges is capped in order to allow smooth rendering in the browser. Equivalent 2D figures are displayed for the same set of network measures, where again the node radius corresponds to the specific measure and the edge width corresponds to edge weight. For each 2D network measure, a bar graph shows the sorted distribution of values for each node in the network.

The analysis also produces figures depicting the thresholded CM with a color bar corresponding to the range of weights in the network. The distribution of node degrees in the network can be assessed with the Node Degree Histogram, which simply bins the degree of each node in the network. Another representation of the network is shown in the Spring Embedded Plot. This diagram collapses the connectivity structure of the network into two dimensions, where each node's “nearness” to each other node is based on the degree of connectivity between them, based on the Fruchterman–Reingold force-directed algorithm implemented in NetworkX. Nodes that are in the same module have the same color.

In order to compare two networks side by side, the networks can be selected on the Compare Networks page. After the two networks have been selected, the same set of options from the Analyze Network page—Weighting Scheme and % of edges to include—must be specified for each network. Once the analysis is run, the same set of measures from the single network analyses are computed for each network. The results are displayed side by side.

Users can assess the impact of a virtual lesion on a network using the Lesion a Brain Network page. Once a network is selected, the user can press “Get Regions” in order to display a checklist of all the brain regions in the given network. Any region that is checked will be “lesioned,” meaning that all of the connections to this node from any other node in the network will be set to zero. The unlesioned and lesioned networks will then be analyzed and the results will be presented side by side in the exact same fashion as the Compare Networks results page.

#### Data sharing

Connectivity matrices can be shared on the Upload New Data page (Figure 2). To share data, the following are required: (1) Study Name, a succinct identifier for the location/purpose of the study, e.g., UCLA_ICBM, (2) Network Name, a succinct name for individual matrix to be uploaded, e.g., CONTROL_grpmean, (3) the uploader's email address, (4) Region Names File, a text file listing the full name of each brain region in the network on separate lines, (5) Region Names Abbreviations File, a text file listing the abbreviated name of each region on separate lines, (6) Region XYZ Centers File, a text file with (X, Y, Z) coordinate for each region (preferably based on mm coordinates in MNI152 space), (7) Connectivity Matrix File, a tab-delimited text file containing the network CM, (8) Imaging Modality, and (9) Share, the sharing status of the data, which can be public (viewable and downloadable by any site visitor) or private (viewable only by the sharer of the data when they are logged in). Other requested meta-information includes the Scanner Device, Scan Parameters, Age Range Minimum and Maximum (the same for an individual subject, different for a group average), Gender, Subject Pool, Group Size, Preprocessing Notes, and Funding Source. Optional fields are also provided for specific imaging parameters for Magnetic Resonance (MR; field strength, MR TR, MR TE, MR Voxel Size, MR Field of View) and data processing steps for fMRI (Motion Correction, Skull Stripping, Temporal Filtering, Spatial Smoothing, Slice Timing Correction, Intensity Normalization, EPI Unwarping, CSF Signal Regression, White Matter Signal Regression, Global Signal Regression), dwMRI (Number of Directions, Maximum b Value, Eddy Correction, Skull Stripping, Deterministic Tractography, Probabilistic Tractography), and structural MRI (sMRI; Skull Stripping, Intensity Normalization).

Data can be shared either for one network at a time or for a set of matrices. In the case of a batch upload, the entries for each of the four required text files simply needs to be stacked vertically for as many networks as will be uploaded. For example, if the network size was 100 × 100, the CM text file for six networks would be 600 rows by 100 columns. For the region names, region abbreviations, and region coordinates files, the list simply needs to be repeated as many times as there are networks. When performing a batch upload, the meta-information need only be entered once and all of the data can be uploaded with a single entry.

**Figure 5:**
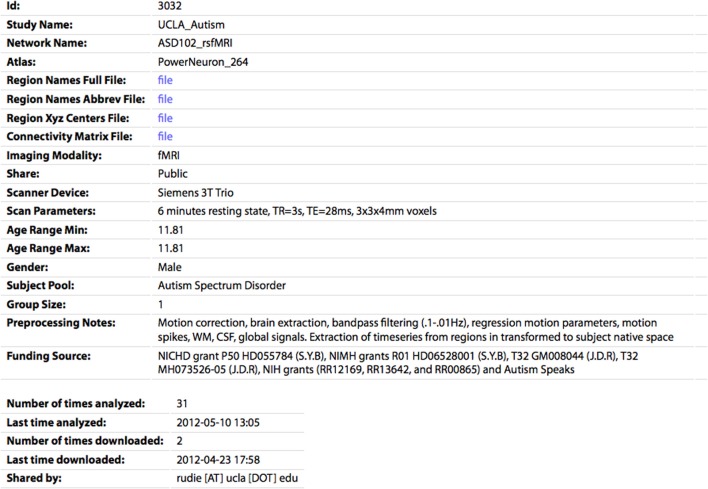
**The profile page for an individual CM.** All of the parameters specified by the sharer of the data appear's on this page. The CM and associated files (region names/abbreviations/XYZ centers) can be downloaded by clicking the “file” links. The number of times the network has been analyzed or downloaded by any user of the site are also shown.

Data on the UMCD can be searched for on the Browse All Data page (Figure 3). Each publicly shared network appears as a row where each column lists a different field describing that entry, as was specified on the Upload Data page when the data was shared. If a user has shared any data privately and is logged in, those data entries will also appear. Any column can be sorted by clicking on the column header, allowing the user to group data by imaging modality or study name, for example. A search box allows the user to enter any term and dynamically display only the rows that contain that term, allowing rapid location of a dataset of interest. Any row has an option to View/Download, linking to an individual “profile” page for the network including the study and network name, the study and subject information, and links to download the raw CM (Figure 5).

If the user is logged in and accessing data they shared, they can also edit or delete the entry on this page. The profile page for each network also shows the number of times the network has been analyzed or downloaded, as a measure of interest the community has in this dataset. On the Browse Data page, each entry also has an Analyze link, which will take the user to the Analyze page with the form prefilled to run the analysis for this network.

Users who wish to download all of the metadata or data for a study can do so on the Browse Studies page. The metadata for a study can be downloaded as either a Comma Separated Value (CSV) or Javascript Object Notation (JSON) file using a URL of the format http://humanconnectomeproject.org/get_study_metadata.<filetype>/<studyname> where “filetype” is one of *csv* or *json* and “studyname” is the study name provided by the individual who shared the data. The connectivity matrices for a given study can be downloaded in a zip file, along with the region names, abbreviations, and XYZ centers, by accessing the URL http://humanconnectomeproject.org/get_study_data/<studyname>.

At the time of writing, the UMCD has 2155 publicly available CMs. These include 1003 functional connectivity MRI (fcMRI) matrices from the 1000 Functional Connectomes sample (Biswal et al., 2010), 522 fcMRI matrices from the ADHD200 sample (http://fcon_1000.projects.nitrc.org/indi/adhd200), 189 DTI matrices from the International Consortium for Brain Mapping dataset (http://www.loni.ucla.edu/ICBM), 175 fcMRI/DTI matrices from a study of autistic children (Rudie et al., under review), 55 DTI matrices from a study of aging and genetic risk for Alzheimer's Disease (Brown et al., 2011), the 392 fcMRI/DTI matrices from the Nathan Kline Institute/Rockland sample on the International Neuroimaging Data-Sharing Initiative site (INDI; including the 60 subjects in the 26–45 age range and another 136 subjects outside that range, from 4 to 85), and a small set of other miscellaneous contributions. All currently available matrices have largely complete metadata including subject demographics, scan parameters, and preprocessing notes. All currently available fMRI matrices are based on Pearson correlation of regional timeseries and all DTI matrices are based on tensor-based deterministic tractography. We anticipate that fMRI and dwMRI matrices from more diverse processing streams will eventually be shared on the site.

#### Interface with neuroscience information framework

In order to facilitate the search of connectivity for specific brain regions in data shared on the UMCD, it is important to interface with various catalogs and wider scope data sharing initiatives. The Neuroscience Information Framework (NIF; http://neuinfo.org) is a project supported by the Blueprint for Neuroscience Research, a pan-NIH initiative with a stated goal of facilitating data discovery and sharing among scientists (Gardner et al., 2008). The NIF works with database partners to gather relevant data and facilitate its' discovery by making data searchable via the user interface and web services. Since September of 2011, public UMCD data has been included in the NIF data index with brain region names aligned to NIF terminology. In order to compensate for brain region naming heterogeneity, regional name synonyms are aligned with the NIF standard ontology (NIFSTD). These data are searchable as a part of the NIF pan-mammalian brain connectivity data set. The human MRI functional/structural connectivity can be viewed alongside rodent and monkey connectivity data (http://neuinfo.org/nif/nifgwt.html?query=nlx_83091). To keep data up to date, NIF “crawls” the UMCD and to find new data on a monthly basis, and curators are prompted to evaluate new data as changes are detected. The current portal and web services are hit more than a million times a month, increasing the possibility of users discovering UMCD data.

### Guidelines for data sharing and analysis

#### Data description and connectivity matrix derivation

To create a connectivity database that can maximize the research and clinical utility of the contributed data, it is essential to first define a set of best practices for deriving CMs. This section will discuss the procedure for deriving CMs from different neuroimaging modalities and the methodological issues that need to be addressed. We will limit the discussion to MRI modalities. For any MRI data shared on the UMCD, the parameters of the scan should be entered in the MR-specific fields and additional details should be noted in the *Scan Parameters* field on the Upload Data page (http://umcd.humanconnectomeproject.org/upload). This generally includes the magnetic field strength, repeat time (TR), echo time (TE), scan duration, field of view, voxel resolution, slice thickness/gap, and other modality-specific factors.

#### fMRI preprocessing

When sharing fMRI-derived connectivity matrices on the UMCD, the user should list check boxes for all included preprocessing steps in the fMRI-specific fields and details should be noted in the *Preprocessing Notes* field. fMRI-specific preprocessing steps include motion correction, linear detrending, smoothing, statistical removal of nuisance variables from white matter (WM), CSF and whole brain signal, and bandpass filtering. For each step, the user should also include which software program was used (FSL, SPM, AFNI, in house, etc.). Although preprocessing methods differ between laboratories, the UMCD does not enforce strict criteria regarding data processing in the interest of remaining open to a maximal number of contributions. Instead, the responsibility is placed with the contributor to ensure that their shared data has been carefully processed, and equally with the site user to use their own discretion for assessing data quality.

When a subject performs a task during fMRI, networks are known to reconfigure to some degree based on the specific cognitive demands of the task (Bassett et al., 2011b; Mennes et al., 2012; Shirer et al., 2012). When task-based functional connectivity matrices are submitted to the UMCD, they should be annotated with a description of the task design and the cognitive processes that the experimenter expected to engage.

#### Diffusion-weighted MRI preprocessing

When sharing dwMRI-derived CMs on the UMCD, the user should specify the scan type as DTI, High Angular Resolution Diffusion Imaging (HARDI), or Diffusion Spectrum Imaging (DSI). In the dwMRI-specific fields, the user should note the number of gradient directions included in the scan sequence along with the maximum *b*-value, whether eddy correction was performed, and the tractography method (deterministic or probabilistic). In the *Preprocessing Notes*, the user should describe the software package used and preprocessing details including whether multiple dwMRI scans were acquired and averaged, how diffusion tensors/Orientation Distribution Functions (ODFs) were calculated, the tractography algorithm, any voxelwise masking criteria, and the maximal angular threshold allowed for fibers to turn between adjacent voxels.

#### Parcellation scheme/choice of atlas

In order to obtain connection strengths between brain regions, the regions must first be defined. This task typically takes one of several routes. Structural parcellation takes a structural image and parcellates it with an algorithm that uses anatomical information in the image and prior models to determine cortical and subcortical regional boundaries. Common analysis packages for performing parcellation are Freesurfer (http://surfer.nmr.mgh.harvard.edu) and Automated Anatomical Labeling (AAL) (Tzourio-Mazoyer et al., 2002). Functional parcellation can be performed on fMRI data based on a search for regions whose functional connectivity patterns are statistically similar in time and/or space (Craddock et al., 2012). A third strategy is to use predefined subregions of cortex/subcortex from a predefined atlas such as the Harvard-Oxford cortical/subcortical probabilistic atlas distributed with the FMRIB Software Library (FSL). The set of ROIs are normally spatially registered to the subject's image space where connectivity is to be estimated. A fourth strategy is to use a set of meta-analytically defined coordinates in a standard stereotactic space (e.g., MNI152) based on sites of peak activation during behavioral tasks. Small regions of interest, typically spheres of 5–10 mm radius, are created around each coordinate and used as seeds to calculate connectivity strength with the remaining spheres (Power et al., 2011). For any parcellation scheme, different tissue types may be included or excluded. fMRI-based analyses are typically uninterested in WM signal and may use gray matter ROIs. Conversely, dwMRI studies are more focused on water diffusion in WM and often use the gray/WM interface as a starting point for tractography. In this case, ROIs may include portions of both gray and WM. For atlas-based ROIs, region boundaries are commonly defined using probabilistic estimates rather than hard cutoffs. In this case, the user must decide a probability threshold above which to assign regional labels.

For submission to the UMCD, the user should note the atlas/software package used to parcellate the brain in the *Atlas* field and any masking operation that was performed for each ROI in the Preprocessing Notes field (e.g., gray matter, WM, probability threshold). For each ROI, the (x, y, z) spatial coordinate describing its spatial location is also required in order to generate network renderings. The spatial center of mass is an ideal descriptive coordinate for an ROI. Users are strongly encouraged to use millimeter coordinates based in the Montreal Neurological Institute coordinate system after registering their ROIs to the MNI152 average brain. This is not strictly enforced and caution is warranted for any site user planning to compare connectivity loci across different datasets on the UMCD.

#### General connectivity matrix preprocessing

Once a CM has been calculated, there are a variety of post-processing strategies that are specific to different imaging modalities, software packages, and laboratories. We urge those who share data on the UMCD to submit “raw” CMs. This precludes thresholding of edge weights below a certain weight cutoff, binarization of edges, or adjustment of weights to deal with issues of non-normal distribution and negative weights. Additionally, all current UMCD analyses assume that CMs are symmetric, meaning that the connection weight from node *i* to *j* is identical to the connection weight from *j* to *i*. The storage of raw data is necessary for subsequent downloaders of the data to make their own decisions about how to treat the data.

#### General connectivity matrix analysis

The only options the user can configure when running an analysis on the UMCD are the weighting scheme and the edge density. All graph measures calculated by the UMCD are interpretable for binary and weighted graphs. While the various arguments for using binary or weighted edges are beyond the scope of this paper, it is generally helpful to test both options when analyzing a network in order to see how different graph metrics will vary. The selected edge density will also affect the resultant graph metrics. At very low edge densities the network is likely to become disconnected, in which case path length-based measures like characteristic path length and lambda cannot be calculated for the graph as a whole. Other measures like global efficiency, clustering coefficient, and modularity can be calculated for disconnected graphs. From an analysis perspective, sparse graphs (with <= ~25% of edges connected) can be considered to have higher “signal to noise” ratio (Alexander-Bloch et al., 2010), preserving only the strongest connections in the network. On the other hand, because the choice of threshold is arbitrary, any chosen threshold may exclude some edges that correspond to true biological connections. Thus, it is important to assess graph metrics across a range of different thresholds to ensure that they are not an artifact of one specific threshold value. For these reasons, users are encouraged to test different permutations of weighting schemes and thresholds to assess how network characteristics may vary.

There are important caveats with a general analysis pipeline like the UMCD. A graph is a very general representation of connectivity strengths in a network model of a system like the brain. Graphs derived from different types of data, such as fMRI and DTI-based graphs, may be more appropriate for certain network measures than others. For example, in functional graphs the edge weights are described based on a statistic, often the correlation coefficient. In this case, path length-based measures such as global efficiency may not be as meaningful as they are in a structural network, where physical connection densities can be measured. The UMCD does not attempt to stratify the networks based on the type of data from which they were derived. It will provide the same complete set of graph theory measures for any analysis. Additionally, most of the graph theory metrics calculated on the UMCD are “unnormalized” with respect to a random network. The only metrics that are calculated as ratios of the true network value to the “pseudo” value from a randomly wired network are gamma (normalized clustering coefficient) and lambda (normalized characteristic path length). Caution is urged to the user in interpreting graph measures to ensure they are used appropriately.

#### Connectivity matrix comparison

In order to compare CMs across studies in a meaningful way, all factors of each study's data collection and analysis must be considered. The imaging modality, scanner, scan sequence, subject pool, and analysis methods will all obviously impact the CM. The regional names used for a given study will also be unique, dependent on the parcellation scheme or atlas that was used to define ROIs. In any attempt to compare CMs from different studies, the user should consider how similar the regions from the studies are. This is a two-pronged issue: first, the spatial position and extent of the ROIs may differ; second, the nomenclature may differ. The UMCD only requests a name and spatial coordinate for each ROI. This is an incomplete description, based on a practical design decision to reduce file storage size and complexity. However, this means that the similarity of ROIs across studies cannot be fully assessed. Each ROI is uploaded with its MNI stereotaxic coordinate, which can establish a coarse measure of spatial similarity in ROI coordinates across studies. The spacing of the full set of coordinates for a given CM can give an idea of the density and average size of each ROI, assuming ROIs are not overlapping. In order to find studies that have connectivity estimates for a given region, the user is encouraged use the NIF interface to the UMCD. There, a user could search for the inferior frontal gyrus, pars triangularis, or Brodmann Area 45, and obtain the same results based on their alignment in the NIFSTD. A more systematic attempt to maximally align all regional names from two different studies is outside the scope of the current work but has been addressed elsewhere (Bug et al., 2008; Imam et al., 2012). Of course, full alignment of ROIs may not be possible if the studies differ in the number of regions, the size of the defined regions, or the set of regions that are excluded (e.g., subcortical nuclei). For all of these reasons, the user must carefully consider how comparable two datasets are, and carry this in mind when comparing data from different studies shared on the UMCD.

### Example analysis

Here we perform an analysis based on publicly available DTI and rs-fMRI data from 60 subjects that were part of the NKI/Rockland study available for download on INDI (http://fcon_1000.projects.nitrc.org/indi/pro/nki.html).

#### Subjects

The purpose of this study was to generate a large scale, extensively phenotyped dataset to explore brain/behavior relationships in healthy individuals. For the current study, we were interested in comparing functional and structural brain networks in healthy adults. We selected 60 subjects from this sample for analysis, ranging in age from 26 to 45 years, mean = 35.8 ± 6.3. Thirty-seven males and 23 females were included. Subjects underwent diagnostic psychiatric interviews, along with a battery of psychiatric, cognitive, and behavioral assessments. Full-scale IQ (FSIQ) was measured with the Wechsler Abbreviated Scale of Intelligence. The mean subject FSIQ was 104.1 ± 12.5.

#### MRI scans

Resting state fMRI was performed on a Siemens Trio 3T with acquisition time = 10:55, TR = 2500 ms, TE = 30 ms, on 38 slices with a voxel size = 3 mm^3^. DTI had acquisition time = 13:32, TR = 10000 ms, TE = 91 ms, on 58 slices with a voxel size = 2 mm^3^ along 64 diffusion-weighted gradients with *b* = 1000 s/mm^2^. A magnetization prepared rapid gradient echo (MPRAGE) scan had either a longer sequence with acquisition time = 10:42, TR = 2500 ms, TE = 3.5 ms on 192 slices with a voxel size = 1 mm^3^ or a shorter sequence with acquisition time = 5:49, TR = 2500 ms, TE = 3.5 ms, on 192 slices with voxel size = 1 mm^3^. The raw data for these scans was accessed from http://fcon_1000.projects.nitrc.org/indi/pro/nki.html.

#### RS-fMRI/DTI processing

Resting state fMRI data was preprocessed using the following pipeline: (1) corrected for differential slice timing using FSL's slicetimer, (2) rigid-body motion corrected each volume to the middle volume using FSL Motion Correction using FMRIB's Linear Image Registration Tool (MCFLIRT), (3) stripped the skull using FSL Brain Extraction Tool (BET), (4) spatially smoothed the data with a Gaussian kernel with 5 mm full-width half maximum, (5) grand-mean scaled the entire 4D dataset, (6) band pass temporal filtered the data from 0.08 to 0.009Hz, (7) performed tissue-type segmentation of the MPRAGE using FSL FAST, (8) registered cerebrospinal fluid (CSF) and WM masks to the first fMRI volume, (9) mask the CSF and WM masks with conservative ventricular and core WM masks derived from the MNI152 atlas, (10) extracted mean timeseries from the core CSF, core WM, and whole brain, (11) constructed a model that included timeseries for core CSF, core WM, whole brain signal, the six motion parameters, and all temporal derivatives, performed linear regression with this model on the data, and obtained the residuals, (12) ran motion scrubbing to identify TRs with a relative motion displacement greater than 0.5 mm or a relative BOLD signal intensity change greater than 0.5% (Power et al., 2012), and (13) registered with FSL FLIRT to the MNI152 average brain with a 4 mm^3^ voxel resolution in a three stage registration from fMRI > initial T2 structural > MPRAGE > MNI152. The residual BOLD data was then analyzed using the spatially constrained spectral clustering method (Craddock et al., 2012) in order to derive 188 gray matter/subcortical/cerebellar ROIs that were spatially contiguous and maximally functionally homogenous across subjects. These ROIs ranged in volume from 28 to 180 voxels. No subjects had more than 100 TRs flagged by motion scrubbing and thus none were dropped from subsequent analysis. An average of 9 ± 17 TRs were flagged for removal. After marking flagged TRs, the mean timeseries for each ROI was calculated and then correlated with all remaining ROI timeseries (excluding flagged TRs) to derive a 188 × 188 functional connectivity MRI (fcMRI) matrix.

DTI data was corrected for motion and eddy current distortions using FSL eddy_correct. The skull was stripped using FSL BET. Diffusion tensors were estimated using Diffusion Toolkit (http://trackvis.org/blog/tag/diffusion-toolkit/) and tractography was run using the fiber assignment by continuous tracking (FACT) algorithm (Mori and van Zijl, 2002), with an angle threshold of 45°. The fractional anisotropy (FA) map for each subject was registered to the MNI152 average brain in a two-stage registration from FA to MPRAGE using a mutual information cost function and 7 degrees of freedom, then from MPRAGE to MNI152 using a correlation ratio cost function and 12 degrees of freedom. The transformation matrices were combined and inverted. We then registered the 188 ROIs defined from the fMRI data in standard space to each subject's DTI space. Masks were dilated by one voxel width in order to include the gray/WM interface, and then thresholded in order to assign each voxel to only the ROI for which it had the highest intensity value (greatest likelihood of membership). For each ROI, all fibers were counted that intersected at least one voxel in the source ROI and at least one voxel in any target ROI using custom code (http://ccn.ucla.edu/wiki/index.php/UCLA_Multimodal_Connectivity_Package). In this way the 188 × 188 structural CM was obtained.

#### Function/structure weight comparisons

Connection weights for functional and structural networks were correlated with each other across subjects using Matlab (The Mathworks, Natick, MA) in order to determine network similarity.

#### Graph theory

All 120 matrices (60 functional + 60 structural) were uploaded to the UMCD. The individual networks are publicly shared on the site under the study name “NKI_Rockland.” The network names are in the format “NKI_<subject_id>_<modality>,” e.g., “NKI_1013090_fcmri” and “NKI_1013090_dti.” For all fcMRI matrices, we also sought to examine the effect of global signal regression (GSR) on functional network topography and similarity to structural networks. GSR is a controversial step in fcMRI preprocessing. Proponents argue that this step controls for common, non-brain sources of variation that affect the entire image (Fox et al., 2009), while opponents have shown that this step may obscure the distribution of connectivity weights and introduce artifactual anticorrelations between networks (Murphy et al., 2009; Saad et al., 2012). We therefore prepared a parallel set of CMs that were processed in identical fashion but without the GSR step. These matrices are labeled as, e.g., “NKI_1013090_fcmri_NoGSR.” From these individual matrices, group-average functional and structural matrices were calculated. These group average matrices increase the stability of connectivity estimates between regions for a relatively homogenous group from a developmental/aging standpoint. The rationale was that individuals in the 26–45 age range are fully mature but have not yet experienced cognitive aging, placing them in the maximally “normal” adult age range. The group averages are named “NKI_fcmri_avg_<age_range_min>_<age_range_max>” and “NKI_dti_avg_<age_range_min>_<age_range_max>.” Finally, in order to test the affect of GSR on the resultant networks, equivalent functional connectivity matrices without the GSR stepped are stored under the name “NKI_fcmri_avg_<age_range_min>_<age_range_max>_NoGSR.”

For the current study, the goal was to demonstrate the capacity of the UMCD to compare functional and structural CMs. Hence, only the NKI_dti_avg_26_45, NKI_fcmri_avg_26_45_GSR, and NKI_fcmri_avg_NoGSR matrices were analyzed. They are henceforth referred to as NKI_dti_avg, NKI_fcmri_avg_GSR, and NKI_fcmri_avg_NoGSR. These matrices were analyzed at two different edge density levels, 5 and 20%. The 5% threshold created sparser matrices that preserved only the strongest edges and highlighted different network modules. The DTI network was disconnected at a 5% threshold, preventing the computation of characteristic path length and small worldness. At the 20% threshold, all networks were fully connected, allowing the computation of global path length-based measures. Matrices were also analyzed with two different weighting schemes, binary and weighted. For functional networks, edge weights spanned a range of −1 to 1, as dictated by the Pearson correlation formula. For structural networks, the fiber connection weights spanned four orders of magnitude (10^0^–10^4^). In cases like these where the distribution of weights for different matrices are significantly different, the binarization of network weights can significantly obscure the underlying connectivity patterns. However, binarization has advantages, including simpler graph theory calculations and a more straightforward randomization scheme (degree preserving rewiring) for determining null reference networks. We therefore found it pertinent to examine both weighted and binarized functional and structural networks.

Each matrix was analyzed and all global/regional measures were downloaded from the UMCD and imported to Matlab. The adjusted Rand index was used to quantify the similarity of modularity partitions in individual functional vs. structural networks, where 0 indicates no agreement between nodes and 1 indicates total agreement between all nodes.

## Results

### Basic graph properties

**Figure 6:**
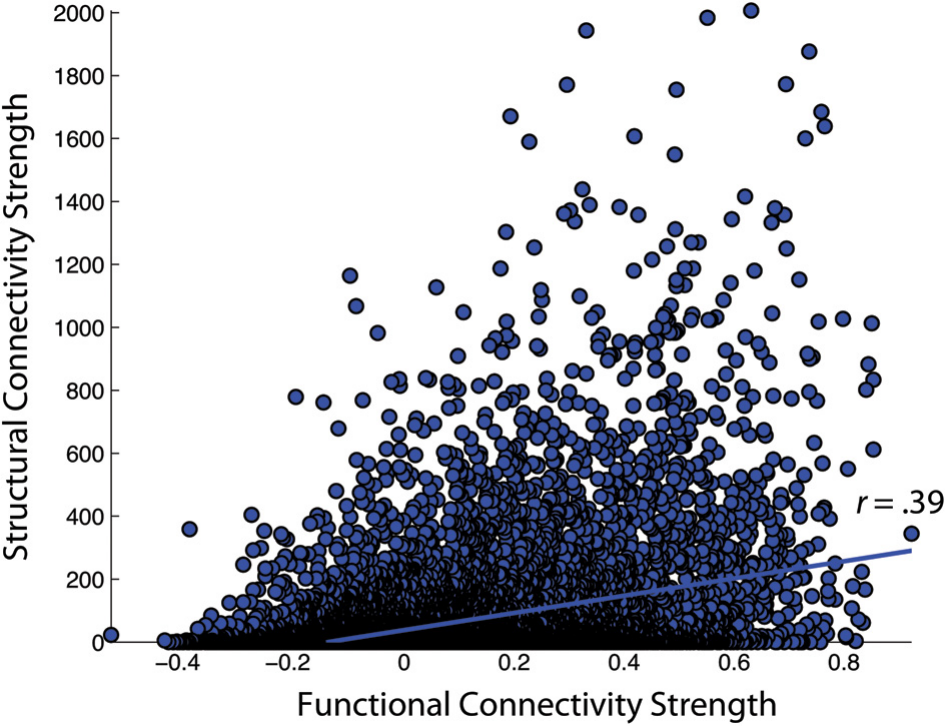
**Correlation of functional and structural connectivity strengths for the group-average 188 × 188 connectivity matrices, identified as NKI_fcmri_avg_GSR and NKI_dti_avg in the text**.

First, the similarity of functional and structural connection weights was assessed by Pearson correlation. For the NKI_fcmri_avg_GSR and NKI_dti_avg CMs, the edge weight correlation was *r* = 0.39 (all *p* < 10^−5^; Figure 6). When only considering regions with existent structural connections (> 1 fiber, averaged across the group), the edge weight correlation increased to *r* = 0.42. For the NKI_fcmri_avg_NoGSR and NKI_dti_avg, the edge weight correlation was *r* = 0.30 considering all connections. When limiting to only existent structural connections, the correlation increased slightly to *r* = 0.34.

### Global graph theory measures

Next, the fcMRI and DTI networks were compared to one another using UMCD's “Compare Networks” feature. The raw NKI_fcmri_avg_GSR network was 100% connected with an average edge weight of 0.017 ± 0.199 (Table 1). The average Euclidean distance between ROI coordinates was 83.5 ± 30.7 mm. The raw NKI_fcmri_avg_NoGSR network was also 100% connected, with a higher average edge weight of 0.28 ± 0.17, as expected. The average Euclidean distance between ROIs was exactly the same as the GSR network, given that every ROI is connected in the raw CMs. The raw NKI_dti_avg network was 76.04% connected with an average edge weight of 56.56 ± 156.10 and an average Euclidean distance of 76.85 ± 29.94 mm.

**Table 1:** **Basic network and global graph theory properties for the fcMRI networks with/without Global Signal Regression and the DTI network**.

**Measure**	**NKI_fcmri_avg_GSR**	**NKI_fcmri_avg_NoGSR**	**NKI_dti_avg**
Raw density	100%	100%	76.04%
Raw edge weights	0.017 ± 0.199	0.28± 0.17	56.56 ± 156.10
Raw Euclidean distance	83.5 ± 30.7 mm	83.5 ± 30.7 mm	76.85 ± 29.94 mm
Thresholded edge weights	0.33 ± 14	0.54 ± 0.1	204.52 ± 249.66
Thresholded Euclidean distance	59.55 ± 30.41 mm	61.19 ± 29.84 mm	50.43 ± 22.74 mm
CPL	2.00	2.06	1.96
MCC	0.57	0.57	0.62
eGlob	0.57	0.56	0.57
Modularity	0.47	0.39	0.33
Small worldness	2.43	1.71	1.81
Gamma	2.72	1.92	1.95
Lambda	1.12	1.12	1.08

Network measures were assessed with binary edges at an edge density of 20% (Table 1). The networks differed from each other for nearly every metric. The measures are listed here in the format (fcMRI GSR/NoGSR vs. DTI). The fcMRI network had greater characteristic path length (CPL; 2.00/2.06 vs. 1.96), lower clustering coefficient (CC; 0.57/0.57 vs. 0.62), equivalent global efficiency (GE; 0.57/0.56 vs. 0.57), higher small worldness for GSR (2.42/1.71 vs. 1.81), higher gamma for GSR (2.72/1.92 vs. 1.95), higher lambda (1.12/1.12 vs. 1.08), and higher modularity (Q; 0.47/0.39 vs. 0.33).

### Nodal graph theory properties

For the nodal measures, the Pearson correlations between functional and structural measures were calculated across the 188 nodes (Table 2). For binarized networks thresholded at 20%, the correlations were significant at a *p*-level of 0.01 for betweenness centrality (*r* = 0.19, *p* = 0.008) and participation coefficient (*r* = −0.2, *p* = 0.005) and nearly significant for clustering coefficient (*r* = 0.18, *p* = 0.016), though the betweenness result was driven by outliers. The correlation was not significant for strength (*r* = −0.06, *p* = 0.45) or regional efficiency (*r* = −0.05, *p* = 0.54). For comparison, nodal measures were also correlated between the functional and structural networks for binary networks thresholded at 5% and weighted networks thresholded at 5 and 20% (Table 2). Each of the measures was also compared using the Spearman rank correlation. In no cases was the Spearman correlation coefficient substantially different from the Pearson correlation coefficient (mean difference in *r*:0.015).

**Table 2:** **Correlation of nodal fcMRI and DTI graph theory measures across all 188 nodes for the NKI_fcmri_avg_GSR and NKI_dti_avg networks**.

	**Binary, 5%**	**Binary, 20%**	**Weighted, 5%**	**Weighted, 20%**
Strength	−0.02 (0.83)	−0.06 (0.45)	−0.02 (0.83)	−0.06 (0.45)
Clustering coefficient	0.09 (0.2)	0.18 (0.016)	0.12 (0.11)	0.11 (0.12)
Betweenness centrality	0.15 (0.036)	0.19 (0.008)^*^	0.14 (0.06)	0.15 (0.04)
Regional efficiency	−0.01 (0.91)	−0.05 (0.54)	−0.1 (0.15)	−0.01 (0.93)
Participation coefficient	0.15 (0.04)	−0.2 (0.005)^*^	0.15 (0.04)	0.04 (0.6)
Adjusted Rand index	0.19	0.09	0.19	0.12

Values are mean r-value (p-value) except for the Rand index.

* Indicates significance at p < 0.01.

**Figure 7:**
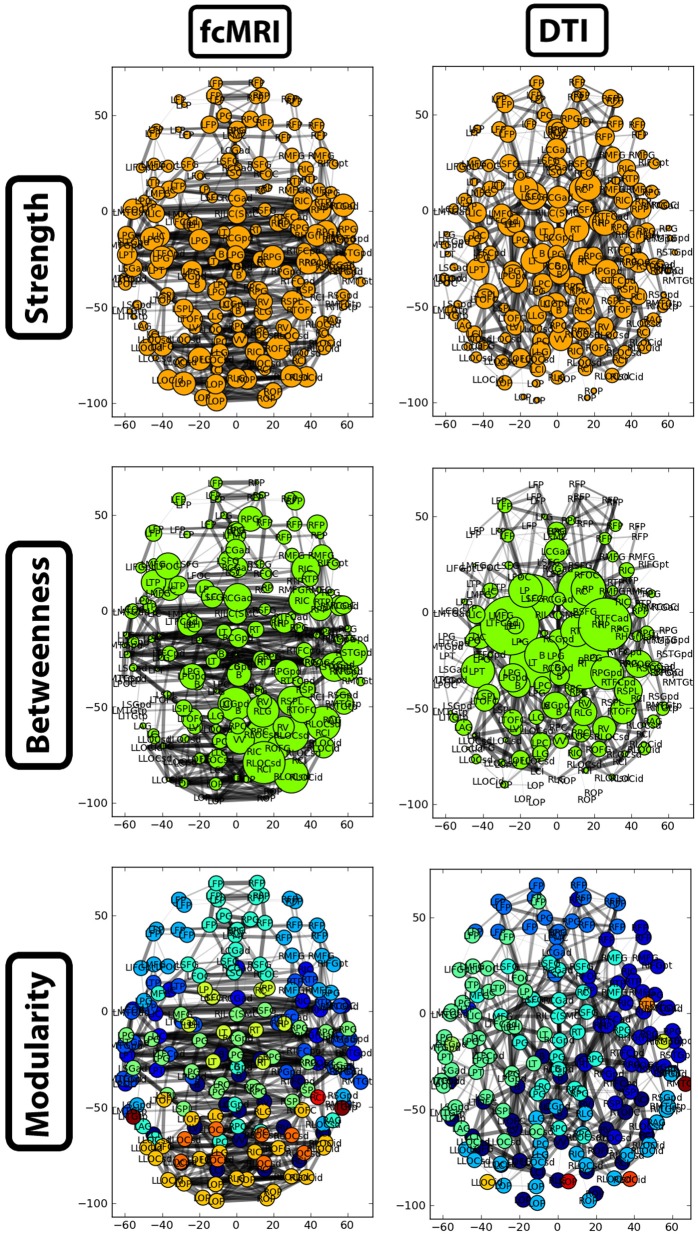
**Two-dimensional network plots from fcMRI and DTI group average networks.** Networks are viewed from a top/axial view with edge width proportional to connection strength and node radius/color related to the given network measure. For each network, the top 4% of weighted edges based on strength are shown. In the first row, the radius of each node is based on its connection strength. In the second row, the radius of each node is based on its betweenness centrality. The third row shows nodes grouped into different modules by color.

**Figure 8:**
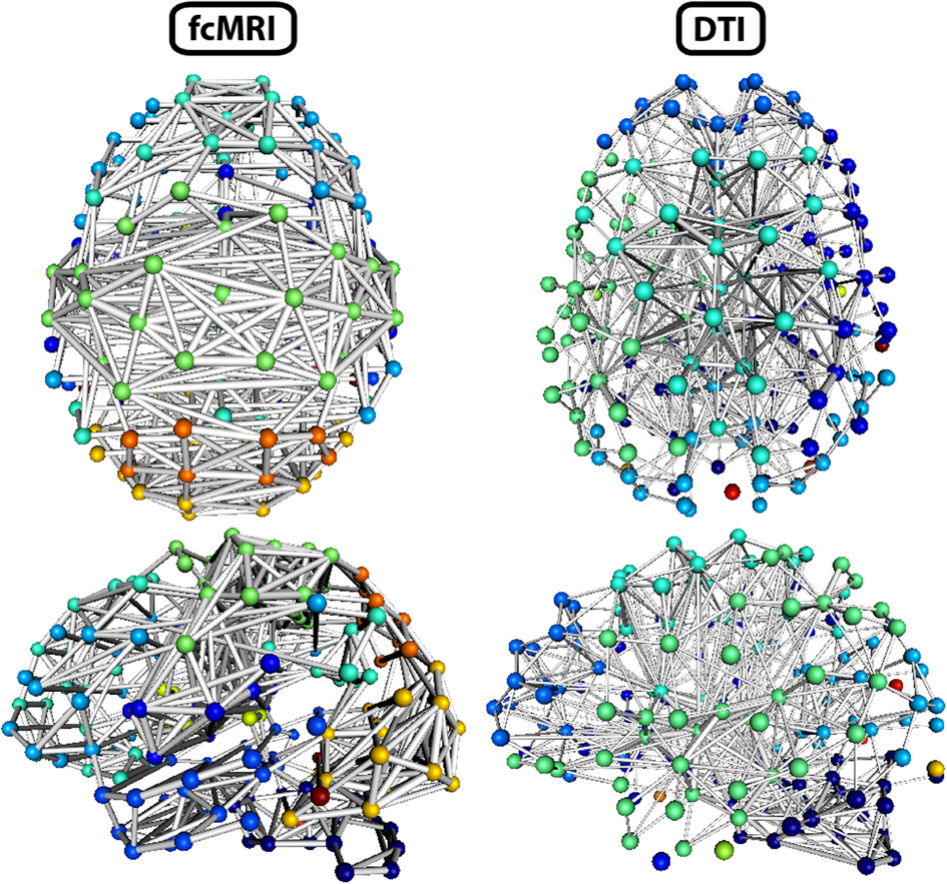
**Three-dimensional network renderings of the fcMRI and DTI group average networks, thresholded to show the top 4% of weighted edges based on connection strength.** Node colors are based on module membership. The same networks are shown from a top view and a left side view. The fcMRI network shows modules agreeing with known functional networks, longer edge Euclidean lengths, and more bilateral connectivity. The DTI network shows more anatomically confined modules, shorter edge Euclidean lengths, and more local connectivity.

A side-by-side visual comparison of the networks revealed the fcMRI network had more bilateral connectivity (Figures 7 and 8), particularly in the motor and visual cortices. The average length of edges between nodes, as measured by Euclidean distance, was longer in the fcMRI network than the DTI network (Table 1). The nodes with the highest “hubness,” based on the combined rank of strength, betweenness centrality, and regional efficiency are shown in Table 3.

**Table 3:** **Regions with the highest combined rank for node strength, betweenness centrality, and regional efficiency, based on fcMRI/DTI networks binarized and thresholded to keep the 20% strongest edges**.

**fcMRI w/global signal regression, Binary 20%**	**fcMRI w/out global signal regression, Binary 20%**	**DTI, Binary 20%**
Right planum polare	Left temporal occipital fusiform gyrus	Left thalamus
Right precuneus	Right temporal occipital fusiform gyrus	Right pallidum
Cerebellum vermis VI	Right anterior cingulate	Right thalamus
Right anterior middle temporal gyrus	Left occipital fusiform gyrus	Left pallidum
Right posterior parahippocampal gyrus	Right temporal pole	Left posterior parahippocampal gyrus
Left posterior cingulate	Right precuneus	Right putamen
Right anterior cingulate	Right superior lateral occipital cortex	Right posterior parahippocampal gyrus
Left posterior middle temporal	Right temporooccipital inferior temporal gyrus	Left insula
Right paracingulate	Right posterior superior temporal gyrus	Left caudate
Right posterior superior temporal gyrus	Right planum polare	Right hippocampus

The functional network with GSR exhibited hubs in the temporal lobe, cingulate cortex, parietal lobe, and cerebellum. Without GSR, hubs were more apparent in occipital and temporal lobes. Structural hubs were found in the subcortical areas (thalamus, caudate, putamen, pallidum), medial temporal lobe, and insula.

The strength, betweenness centrality, and modularity of each node for the NKI_fcmri_avg_GSR and NKI_dti networks, weighted and thresholded at 4% for visualization purposes, are displayed visually on the 2D network in Figure 7. fcMRI network nodes with high strength and betweenness centrality were spatially distributed while in the DTI network they tended to cluster in the subcortical nodes. The fcMRI modules corresponded to well-characterized functional systems including the default mode network (turquoise), temporal lobe network (blue), fronto-parietal network (light blue), dorsal and ventral sensory-motor networks (blue and seafoam green), visual network (yellow), posterior parietal network (orange), subcortical network (line green), and cerebellar network (dark blue) (Power et al., 2011; Tomasi and Volkow, 2011) (Figure 8). DTI modules corresponded to neighboring anatomical regions including the frontal lobe/anterior midline (blue), left temporal lobe/parietal lobe/subcortex (seaform green), right temporal lobe/parietal lobe (blue), right central midline/sensory-motor cortex/cingulate cortex (turquoise), occipital lobe/right posterior parietal/temporal lobe (light blue), and cerebellum (dark blue).

## Discussion

### Data sharing and neuroinformatics

The UMCD allows any user to publicly share brain connectivity matrices, run graph theory-based analyses on the website, and search available data across any imaging modality, demographic category, or disease status. The ability to archive these data in their complete form and make them publicly available should enable more extensive brain connectivity meta-analyses. Here we illustrated the capability of the UMCD to compare functional (fMRI) and structural (DTI) CMs derived from the same set of subjects in order to assess similarities and differences in their connectivity patterns.

The UMCD decreases the barrier to entry for performing a graph-theory based analysis of a CM. We emphasize that this platform is not primarily designed for statistical analysis. It is first and foremost a data sharing site. We provide graph theory based tools to allow users to explore and compare CMs of interest on the site. While we consider the richness of this environment to be beneficial, caution is warranted in the application and interpretation of graph theory measures from UMCD. As with any software package that provides quantitative data metrics, users are urged to thoroughly consider how these measures were calculated and whether they are appropriate to compare across individual, imaging modality, or study.

MRI-based connectivity analyses offer hope in improving the ability of a clinician to diagnose a neurological disease or neuropsychiatric disorder. In order to achieve accurate diagnosis of individual patients, sensitivity and specificity of classification must be pushed to extremely high levels (Pepe et al., 2004). One obvious way to improve classification accuracy is to increase the number of training samples. Community-driven repositories are an effective strategy for rapidly aggregating large amounts of data from the international community (van Horn et al., 2004; Milham, 2012). These repositories have already been leveraged to build neuroimaging-based classifiers of neuropsychiatric disorders such as Attention Deficit Hyperactive Disorder and evaluate their efficacy (Cheng et al., 2012; Colby et al., 2012; Eloyan et al., 2012). A downside of this strategy is the increased likelihood of data with suspect quality. While users are encouraged to share data on the UMCD only after publication, this is not strictly enforced. We leave the accessors of the data the responsibility of vetting data that they analyze on the UMCD or download for off-site use.

One aim of the neuroinformatics field is the integration of databases with one another into large federations (Akil et al., 2011). These efforts are dependent on the establishment of ontologies and the use of application programming interfaces (APIs). The NIF is a semantic search engine that allows a user to search a broad set of databases spanning many species, recording methods, and laboratories (Gupta et al., 2008). The UMCD connectivity data are regularly crawled by NIF as they are uploaded to the system, using the region names associated with each shared CM. This allows a NIF user to perform a connectivity-based meta-analysis at a broader scale. This is a challenging task because data from the various source databases catalogued by NIF are often customized to a particular technique and a particular species, rather than across species and techniques. While mapping Brodmann areas to corresponding cortical structures in rodents may be an ill-posed problem, even simple differences such as “cornu ammonis 1” vs. “Hippocampal region, CA1” present a problem when comparing connectivity measures in different datasets. Note, these are two perfectly valid ways to describe the CA1 region and yet no computer will be able to find these terms together because they are not lexical variants unless the computer is told that these are in fact synonyms. Therefore, NIF superficially aligns brain region labels to the NIF standard ontology, the NIFSTD, where labels can be toggled for search and browsing. NIF also performs a search across all known synonyms per brain region, as it is unlikely that all data will be aligned at any one time. If users wish to investigate connectivity for a specific brain region based on data in the UMCD, they should first perform a search on the NIF system for that region. Once relevant datasets have been identified, they can use the UMCD to further probe the connectivity of their region of interest in those datasets.

### Comparison of functional and structural connectivity matrices

As a demonstration of the UMCD platform, we compared group-averaged functional and structural connectivity matrices from a group of 60 healthy subjects aged 26–45. Functional and structural graph theory-based studies have expanded in parallel in recent neuroimaging literature. Several studies have made direct comparisons of connectivity strengths in rs-fMRI and dwMRI data (Hagmann et al., 2008; Honey et al., 2009) but to our knowledge, none have systematically compared graph-theory based measures. We found that despite a positive correlation of functional and structural connectivity strengths, there was a low correspondence of global and nodal graph theory measures. For the connection weights of fcMRI and DTI networks, the correlation was moderate but statistically significant. The fcMRI network had greater characteristic path length (CPL; 2.00/2.06 vs. 1.96), lower clustering coefficient (CC; 0.57/0.57 vs. 0.62), equivalent global efficiency (GE;0.57/0.56 vs. 0.57), higher small worldness for GSR (2.42/1.71 vs. 1.81), higher gamma for GSR (2.72/1.92 vs. 1.95), higher lambda (1.12/1.12 vs. 1.08), and higher modularity (Q;0.47/0.39 vs. 0.33). The differences in modularity highlight the differences between these networks. The higher modularity in the fcMRI networks relates to the longer path length, as fcMRI networks are more spatially distributed. The DTI network has a more regular, lattice-like topology. Importantly, GSR exaggerates the differences between the fcMRI and DTI networks. Modularity and small worldness both increase drastically in fcMRI data with GSR applied, exaggerating the differences in global graph theory measures to between the fcMRI and DTI networks. This step may enhance within-module correlations while dampening between-module correlations. Surprisingly, the weight correlations for the fcMRI and DTI networks were more similar with GSR applied. Thus, while individual functional and structural weights are more similar after GSR, global network properties become more dissimilar.

DTI and fMRI have their own limitations for determining connectivity strengths. A tensor is a basic model of water diffusion that is insufficiently complex for describing the intersection of multiple fiber populations within a voxel (Wedeen et al., 2008). DTI tractography therefore has limited ability to detect crossing fibers. Alternative diffusion weighted imaging methods like DSI collect data with more gradient directions and larger b-values. This enables the modeling of diffusion as a more complex ODF, which can better resolve the intravoxel crossing of fiber bundles. The corpus callosum can be difficult to fully resolve using DTI because of its crossing with the heavily myelinated corona radiata. Here we derived connectivity matrices from DTI tractography and thus may have slightly underestimated interhemispheric connectivity. Meanwhile, rs-fMRI functional connectivity robustly detects bilaterally symmetric functional connections (e.g., Damoiseaux et al., 2006). Future studies with DTI data, DSI data, and resting state fMRI data will be required to determine how variable the DTI/DSI interhemispheric connectivity measures are, and how these both relate to rs-fMRI connectivity strengths.

The comparison of nodal measures from functional and structural networks revealed mostly non-significant correlations. Importantly, the relationship tended to be non-significant regardless of the weighting scheme or weight threshold, suggesting that these factors do not highly influence the network measures. The only significant finding was a negative correlation of nodal participation coefficients in binary networks at a 20% weight threshold. In order to probe the relationship deeper, we examined the effects of all structural regional measures—degree, clustering, betweenness, regional efficiency, and participation—on functional participation using hierarchical regression. The most significant model for predicting functional participation included structural clustering (*p* = 3.15 × 10^−6^) and participation (2.61 × 10^−7^), both with negative coefficients. Thus, for binarized graphs thresholded at 20%, low structural diversity and sparse local connectivity related to higher functional diversity. A structural “connector” region is one that is sparsely connected and bridges between different areas of the network. In these networks, structural connector regions tended to functionally interact with multiple different functional modules.

Functional and structural modules had a low degree of correspondence in these networks. One explanation for the disparity between these network types is simply that brain structure and function are not isomorphic (Deco et al., 2011). While the structural connective backbone does provide the scaffolding upon which neuronal communication occurs, it may not substantially constrain the functional integration and segregation of brain networks. Structural fiber connections do exist between most intrinsic functional connectivity networks (van den Heuvel et al., 2009a) but such connections do not necessarily imply a similar community structure. Resting state functional connectivity patterns have relatively high test–retest reliability across sessions, indicative of a stable resting state configuration (Shehzad et al., 2009). However, it has been demonstrated that nodes of the brain's functional network exhibit characteristic macroscale reconfigurations in the service of motor tasks (Bassett et al., 2011b), visual perception (Ekman et al., 2012), and episodic memory (Shirer et al., 2012). It appears that certain brain regions may be more predisposed for task-related adaptation while others maintain more stable roles maintaining intrinsic connectivity (Mennes et al., 2012). Meanwhile, the brain's structural macroscale connectivity is known to be largely static and reproducible on short timescales (days to weeks) (Bassett et al., 2011a; Cammoun et al., 2012). The presence of an adaptive functional network on a static structural scaffold obviously indicates some divergence of structural and functional network properties. The aggregation of functional connectivity matrices across resting state and different tasks, when collected in parallel with structural connectivity matrices, should further our understanding of the constraints that structural connectivity places on functional integration and segregation.

## Conclusions

Here we introduced the UCLA Multimodal Connectivity Database, a web-based resource that is openly available for brain network analysis and data sharing. Within this framework, a user can share CMs derived from neuroimaging data or access the matrices that have been publicly shared by other users. The site allows the user to conduct a graph theory analysis of any shared CM and view a report of global and nodal graph theory metrics, 3D and 2D network visualizations, along with study/demographic information about the network. We hope that this website will encourage broader sharing of CMs, enabling large-scale meta-analyses of brain connectivity.

### Conflict of interest statement

The authors declare that the research was conducted in the absence of any commercial or financial relationships that could be construed as a potential conflict of interest.
